# Dual specificity phosphatase 5 and 6 are oppositely regulated in human skeletal muscle by acute exercise

**DOI:** 10.14814/phy2.13459

**Published:** 2017-10-09

**Authors:** Shirin Pourteymour, Marit Hjorth, Sindre Lee, Torgeir Holen, Torgrim M. Langleite, Jørgen Jensen, Kåre I. Birkeland, Christian A. Drevon, Kristin Eckardt

**Affiliations:** ^1^ Department of Nutrition Institute for Basic Medical Sciences Faculty of Medicine University of Oslo Oslo Norway; ^2^ Diabetes and Metabolism Division Garvan Institute of Medical Research Darlinghurst New South Wales Australia; ^3^ Department of Physical Performance Norwegian School of Sport Sciences Oslo Norway; ^4^ Department of Endocrinology Morbid Obesity and Preventive Medicine Oslo University Hospital and University of Oslo Oslo Norway

**Keywords:** DUSP, human exercise study, MAP kinase phosphatase, skeletal muscle

## Abstract

Physical activity promotes specific adaptations in most tissues including skeletal muscle. Acute exercise activates numerous signaling cascades including pathways involving mitogen‐activated protein kinases (MAPKs) such as extracellular signal‐regulated kinase (ERK)1/2, which returns to pre‐exercise level after exercise. The expression of MAPK phosphatases (MKPs) in human skeletal muscle and their regulation by exercise have not been investigated before. In this study, we used mRNA sequencing to monitor regulation of MKPs in human skeletal muscle after acute cycling. In addition, primary human myotubes were used to gain more insights into the regulation of MKPs. The two ERK1/2‐specific MKPs, dual specificity phosphatase 5 (DUSP5) and DUSP6, were the most regulated MKPs in skeletal muscle after acute exercise. *DUSP5* expression was ninefold higher immediately after exercise and returned to pre‐exercise level within 2 h, whereas *DUSP6* expression was reduced by 43% just after exercise and remained below pre‐exercise level after 2 h recovery. Cultured myotubes express both MKPs, and incubation with dexamethasone (Dex) mimicked the in vivo expression pattern of *DUSP5* and *DUSP6* caused by exercise. Using a MAPK kinase inhibitor, we showed that stimulation of ERK1/2 activity by Dex was required for induction of *DUSP5*. However, maintaining basal ERK1/2 activity was required for basal *DUSP6* expression suggesting that the effect of Dex on *DUSP6* might involve an ERK1/2‐independent mechanism. We conclude that the altered expression of *DUSP5* and *DUSP6* in skeletal muscle after acute endurance exercise might affect ERK1/2 signaling of importance for adaptations in skeletal muscle during exercise.

## Background

Physical activity is beneficial for health, and may prevent development of many chronic diseases like obesity and type 2 diabetes (Knowler et al. 2002; Catenacci and Wyatt 2007). The effects of exercise on health and the study of underlying mechanisms have gained much interest because of its marked potential to combat the global epidemic of obesity and type 2 diabetes (Pedersen and Saltin 2015). Physical activity promotes specific reactions and adaptations in most tissues of the body including skeletal muscle. The effects depend on variables like duration, intensity, and type of exercise (Egan and Zierath 2013). Long‐term training causes changes in contractile proteins, mitochondrial function, capillary density, and oxidative capacity along with altered expression of proteins involved in several metabolic and signaling pathways (Hawley et al. 2014). These long‐term adaptations to exercise are due to repeated bouts of muscle contraction. A single bout of exercise activates numerous signal transduction cascades and promotes changes in mechanical strain, ATP turnover, calcium flux, and production of reactive oxygen species (Egan and Zierath 2013). One of the pathways elicited by acute exercise is the mitogen‐activated protein kinase (MAPK) pathway involving p38 MAPK, c‐Jun N‐terminal kinases (JNK), and extracellular signal‐regulated kinase (ERK) 1/2 (Aronson et al. 1997; Widegren et al. 2000). In human skeletal muscle, ERK1/2 phosphorylation is increased in an intensity‐dependent manner by acute contractions. After exercise this phosphorylation is rapidly reduced, and resting levels are restored within 60 min (Krook et al. 2000; Widegren et al. 2000).

MAPKs including ERK1/2 are dephosphorylated by MAPK phosphatases (MKPs) on both the threonine/serine and tyrosine residues. MKPs belong to a subfamily of the dual specificity phosphatases (DUSPs) family and include 10 members, which differ in their substrate specificity, subcellular localization, tissue expression, and inducibility by extracellular stimuli (Camps et al. 2000). Among the MKPs, DUSP5 and DUSP6 (also known as MKP‐3) are specific to ERK1/2 (Arkell et al. 2008; Kucharska et al. 2009) and display specific cellular distribution. DUSP5 is located in the nucleus (Mandl et al. 2005), whereas DUSP6 is found in the cytoplasm (Groom et al. 1996; Muda et al. 1996). Both DUSP5 and DUSP6 sequester unphosphorylated ERK1/2 in the nucleus and cytoplasm, respectively (Karlsson et al. 2004; Mandl et al. 2005). In mammalian cells like NIH 3T3 fibroblasts, some stimuli such as growth factors and lipopolysaccharide (LPS) regulate expression of DUSP5 and DUSP6 via ERK activity (Ekerot et al. 2008; Kucharska et al. 2009).

The expression and regulation of both ERK1/2‐specific DUSP5 and DUSP6 in human skeletal muscle have not been investigated. In this study, we focus on the expression of different MKPs in human skeletal muscle as well as adipose tissue, and their regulation by exercise. We observed that acute exercise altered the expression of most MKPs, where *DUSP5* and *DUSP6* showed the most prominent alteration and were oppositely regulated. We observed that acute stimulation with dexamethasone (Dex) can mimic this in vivo pattern of *DUSP5* and *DUSP6* expression and provide evidence for the involvement of ERK1/2 signaling in regulation of these MKPs in primary human myotubes.

## Methods

### Human exercise intervention study

**Figure 1:**
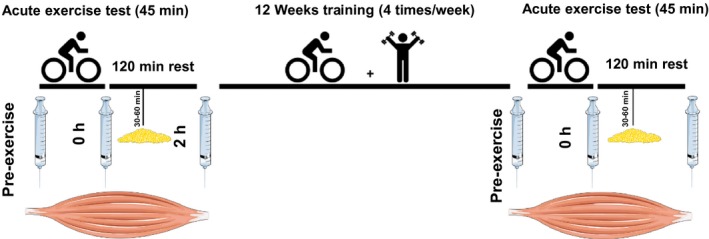
Design of 12 weeks exercise intervention study. Skeletal muscle biopsies were harvested pre‐exercise (pre), immediately after (0 h), and 2 h after an acute (45 min) bicycle sessions, both at baseline and after 12 weeks of exercise intervention. Adipose tissue biopsies were taken 30–60 min after the acute exercise at baseline as well as after 12 weeks of exercise intervention.

Sedentary men (age 40–65 years) participated in a training intervention study described in detail by Langleite et al. (2016). Briefly, men in the control group had a mean body mass index (BMI) of 23.5 ± 2.0 kg/m^2^ (*n* = 13) and normal fasting and 2 h serum glucose levels. In the dysglycemic group, the mean BMI was 28.9 ± 2.5 kg/m^2^ (*n* = 11) and the participants had fasting glucose concentration ≥5.6 mmol/L and/or impaired oral glucose tolerance test (2 h serum glucose concentration ≥7.8 mmol/L). All participants were subjected to a combined strength and endurance training program for 12 weeks (long‐term exercise), including two endurance bicycle sessions (60 min each) and two whole‐body strength training sessions (60 min each) per week. In addition, the participants performed an acute exercise test (45 min cycling at 70% *V*O_2_max, after 10 min warm up) before as well as after the long‐term exercise (Fig. 1). The study was registered with the U.S. National Library of Medicine Clinical Trials registry (NCT01803568).

Biopsies from *m. vastus lateralis* were taken pre‐exercise, immediately after, and 2 h after completing the acute exercise bout. Moreover, adipose tissue biopsies were taken 30–60 min after completing the acute exercise bout. The samples were taken both at baseline and after 12 weeks of exercise intervention, and in total, six biopsies from skeletal muscle and two from periumbilical subcutaneous adipose tissue were obtained during the study from each participant. From one subject, the 2 h samples are missing both at baseline and after 12 weeks. The tissue samples were immediately transferred to RNA‐later (Qiagen, Limburg, Netherland), kept overnight at 4°C, and transferred to −80°C until further processing.

### High throughput mRNA sequencing

RNA was isolated from biopsies of skeletal muscle and adipose tissue, reverse transcribed into cDNA and deep sequenced (Li et al. 2014). In brief, an Illumina HiSeq 2000 platform with multiplex design was used to amplify and deep sequence sonicated cDNA fragments with 51 bp nucleotides. Tophat 2.0.8 with Bowtie 2.1.0 was used with default settings (Langmead et al. 2009; Kim et al. 2013) to align the RNA‐seq reads against the UCSC hg19 annotated transcriptome and genome. Gene filtering and normalization were performed with EdgeR v3.4.2 (Robinson et al. 2010) using a negative binominal generalized linear model in R v3.0.3 (The R Foundation, Vienna, Austria). Normalized gene expression levels are presented in fragments per kB mapped reads (FPKM).

### Culture of primary human skeletal muscle cells

Biopsies from either *m. obliquus internus abdominis* or *m. vastus lateralis* of three male donors (age 33–62 years) were used to isolate primary satellite cells (Haugen et al. 2010). In addition, human satellite cells from one female donor were obtained from Lonza (Basel, Switzerland). The cells were subcultured using SkGM Bullet kit (Lonza) containing 2% fetal bovine serum (FBS) to establish stocks of cells in passage 5. For experiments, myoblasts were seeded in cell culture dishes and proliferated in growth medium (DMEM/F12 HAM 1:1 [Gibco, Life Technologies, Grand Island, NY; Sigma‐Aldrich, St. Louis, MO]) containing GlutaMAX^™^ (Gibco, Life Technologies, Paisley, UK), 50 U/mL penicillin, 50 g/mL streptomycin, 10% FBS, 1 nmol/L insulin, 10 ng/mL epidermal growth factor, 2 ng/mL basic fibroblast growth factor, and 0.4 mg/mL Dex (Sigma‐Aldrich). When the cells were 80% confluent, differentiation was initiated by switching from growth medium to differentiation medium (DMEM/F12 HAM 1:1 containing GlutaMAX^™^, 50 U/mL penicillin, 50 g/mL streptomycin, and 2% horse serum [Sigma‐Aldrich]) for 5 days to obtain multinucleated myotubes. Prior to final incubations, medium was changed to serum‐free differentiation medium for 16 h. Then Dex (0.5 *μ*mol/L) was added to the cells for the indicated times. MAPK kinase (MEK) inhibitor PD98059 (PD, 10 *μ*mol/L, Cell Signaling Technology, Danvers, MA) was added to the cells for 1 h prior to Dex. Because the stock solution of PD was prepared in dimethyl sulfoxide (DMSO), controls containing 0.1% DMSO were included in all experiments involving the inhibitor. No difference was found between control cells and cells incubated with 0.1% DMSO.

### Quantitative real‐time PCR

Total RNA was isolated from cell cultures using the RNeasy Mini kit (Qiagen, Hilden, Germany) according to the manufacturer's protocol. RNA was reverse transcribed into cDNA using High Capacity cDNA Reverse Transferase Kit (Thermo Fisher, Foster City, CA), and RNA expression was analyzed by quantitative real‐time PCR using TaqMan probes and predesigned primers (Thermo Fisher). The following targets were analyzed: *DUSP5* (Hs00244839_m1), *DUSP6* (Hs04329643_s1), *MYH2* (Hs00430042_m1), and *MYOG* (Hs00231167_m1). Expression of the housekeeper gene large ribosomal protein P0 (*RPLP0*, Hs99999902_m1) was used for normalization, and CT values were calculated using the ΔΔCT method.

### Immunoblotting

Cells were lysed in a buffer containing 50 mmol/L HEPES (pH 7.4), 1% Triton X‐100 (v/v), complete protease inhibitor cocktail, and PhosSTOP phosphatase inhibitor cocktail (both from Roche Diagnostics, Basel, Switzerland), followed by pulse‐mode ultrasonication. Lysates were centrifuged at 10,000 *g* for 15 min; supernatants were transferred to fresh tubes and protein concentrations were determined. Equal amounts of protein were loaded to SDS‐PAGE using 4–20% Criterion XT Bis‐Tris precast gels (Bio‐Rad, Hercules, CA) followed by transfer onto polyvinylidene fluoride membrane (Immobilon‐P; Millipore, ‎Billerica, MA). TBST‐T blocking buffer containing 20 mmol/L Tris pH 7.5, 137 mmol/L NaCl, 0.1% Tween‐20 (v/v), and 3% bovine serum albumin (w/v) was used to block nonspecific protein binding by incubating the membrane for 1 h at room temperature. Incubation with primary antibodies was performed in the same buffer overnight at 4°C. Antibodies directed against phospho‐ERK1/2 (Thr202/Tyr204) (Cell Signaling Technology), total ERK1/2 (Cell Signaling Technology), and tubulin (Millipore) were used. Peroxidase‐conjugated anti‐rabbit IgG was used as secondary antibody. The SuperSignal West Dura Extended Duration Substrate from Thermo Fisher Scientific was used for enhanced chemiluminescence detection. The signals were visualized and evaluated on a ChemiDoc Touch Imaging System (Bio‐Rad).

### Presentation of data and statistics

Data are presented as means ± SEM. Statistical significance was determined by Student's *t*‐test or one‐way ANOVA (post hoc test Tukey's multiple comparison test) using GraphPad Prism 6 software, and *P* < 0.05 was considered statistically significant.

## Results

### Expression of MKPs in human skeletal muscle and adipose tissue

**Figure 2:**
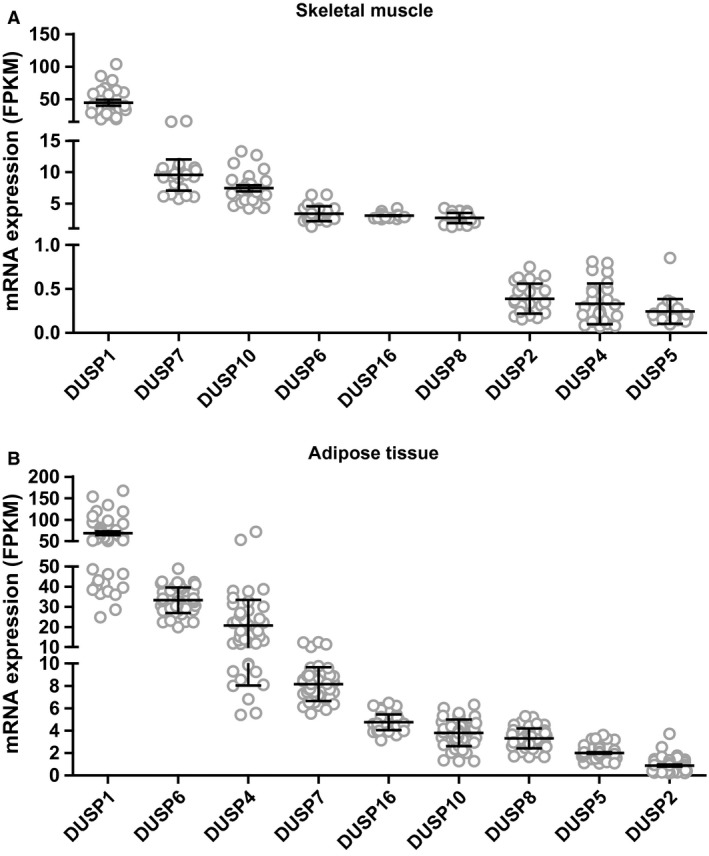
Baseline mRNA expression of MAPK phosphatases (MKPs) in biopsies from (A) skeletal muscle and (B) adipose tissue. Data represent means ± SEM; circles indicate individual expression levels of all participants (*n* = 24). MAPK, mitogen‐activated protein kinases.

mRNA sequencing of skeletal muscle and adipose tissue biopsies revealed that nine of 10 members of the MKP family were expressed in both tissues (Fig. 2). The expression level of different MKPs varied between 45 ± 4.6 FPKM (*DUSP1*) and 0.25 ± 0.03 FPKM (*DUSP5*) in skeletal muscle, and 68 ± 4.4 FPKM (*DUSP1*) and 0.87 ± 0.09 FPKM (*DUSP2*) in adipose tissue, which means that DUSP1 was the most abundantly expressed MKP in both tissues. *DUSP1*,* DUSP2*,* DUSP4*,* DUSP5*, and *DUSP6* were more highly expressed in adipose tissue compared with skeletal muscle, whereas *DUSP7*,* DUSP8*,* DUSP10*, and *DUSP16* exhibited similar expression in both tissues.

For most of the MKPs there were no differences between the control and dysglycemic group at baseline (Table 1). However, *DUSP4* expression was significantly lower in skeletal muscle of the control group, whereas its expression in adipose tissue was higher in the control group. Moreover, a higher expression of *DUSP10* in adipose tissue of dysglycemic men was observed.

**Table 1:** mRNA expression of MKPs in biopsies from human skeletal muscle and adipose tissue at baseline

MKPs	Alternative name	Skeletal muscle		Adipose tissue	
		Control	Dysglycemic	Control	Dysglycemic
DUSP1	MKP‐1	48.41 ± 12.48	51.43 ± 7.52	63.03 ± 10.15	68.67 ± 7.30
DUSP7	PYSr2	9.42 ± 0.69	9.94 ± 0.76	8.64 ± 0.54	8.06 ± 0.27
DUSP10	MKP‐5	6.64 ± 0.35	7.91 ± 0.99	3.13 ± 0.34	**4.32 ± 0.31**
DUSP6	MKP‐3	3.20 ± 0.26	3.30 ± 0.37	35.78 ± 1.93	30.98 ± 1.86
DUSP16	MKP‐7	3.03 ± 0.11	3.13 ± 0.10	4.67 ± 0.20	4.56 ± 0.22
DUSP8	hVH‐5	2.67 ± 0.26	2.83 ± 0.20	2.89 ± 0.31	3.51 ± 0.20
DUSP2	PAC1	0.38 ± 0.05	0.35 ± 0.03	0.85 ± 0.27	1.07 ± 0.17
DUSP4	MKP‐2	0.22 ± 0.03	**0.38 ± 0.08**	25.43 ± 3.46	**13.39 ± 1.43**
DUSP5	VH3	0.27 ± 0.05	0.22 ± 0.02	1.94 ± 0.17	1.89 ± 0.15

Comparison of MKPs expression between the control (*n* = 11) and dysglycemic group (*n* = 13) at baseline in biopsies from human skeletal muscle and adipose tissue. Values represent means FPKM ± SEM; *P*‐values were calculated by unpaired Student's *t*‐test, *P* < 0.05 versus control group (bold). MKPs, MAPK phosphatases; DUSP, dual specificity phosphatase; FPKM, fragments per kB mapped reads.

### Acute exercise enhanced *DUSP5* and reduced *DUSP6* mRNA expression in skeletal muscle

At baseline, the mRNA expression of *DUSP1*,* DUSP2*,* DUSP4*,* DUSP8*,* DUSP10*, and *DUSP16* was increased immediately after acute exercise compared to pre‐exercise (Table 2). Expression of *DUSP2*,* DUSP4*, and *DUSP16* remained elevated 2 h after exercise, *DUSP8* mRNA expression returned to pre‐exercise level within the 2 h recovery period, whereas *DUSP1* and *DUSP10* mRNA expression were significantly lower (~70% and 50%, respectively) 2 h after exercise as compared to pre‐exercise values. Moreover, mRNA expression of *DUSP7* was unaltered just after exercise and 30% reduced after 2 h recovery. When comparing the expression of DUSPs between controls and dysglycemic subjects at 0 h and 2 h, we found no difference at baseline as well as after 12 weeks exercise intervention.

**Table 2:** Regulation of mRNA expression of MKPs in human skeletal muscle at baseline after acute exercise

MKPs	0 h versus pre‐exercise			2 h versus pre‐exercise		
	All	Control	Dysglycemic	All	Control	Dysglycemic
DUSP5	**10.85 ± 1.28**	**11.18 ± 1.72**	**10.45 ± 2.00**	1.25 ± 0.14	1.34 ± 0.25	1.16 ± 0.13
DUSP2	**6.77 ± 1.48**	**7.45 ± 2.29**	**5.96 ± 1.87**	**8.31 ± 0.94**	**9.36 ± 1.58**	**7.16 ± 0.90**
DUSP1	**4.00 ± 0.55**	**4.45 ± 0.79**	**3.47 ± 0.76**	**0.41 ± 0.07**	**0.39 ± 0.07**	**0.44 ± 0.09**
DUSP8	**2.19 ± 0.19**	**2.42 ± 0.31**	**1.91 ± 0.15**	1.23 ± 0.11	1.26 ± 0.19	1.19 ± 0.11
DUSP4	**1.97 ± 0.30**	**2.16 ± 0.42**	1.74 ± 0.45	**2.36 ± 0.34**	**2.73 ± 0.55**	1.96 ± 0.38
DUSP16	**1.51 ± 0.05**	**1.52 ± 0.07**	**1.50 ± 0.08**	**1.15 ± 0.04**	**1.22 ± 0.05**	1.09 ± 0.07
DUSP10	**1.23 ± 0.07**	**1.20 ± 0.07**	1.26 ± 0.13	**0.56 ± 0.05**	**0.59 ± 0.09**	**0.51 ± 0.05**
DUSP7	0.99 ± 0.04	1.00 ± 0.06	0.97 ± 0.04	**0.72 ± 0.04**	**0.74 ± 0.07**	**0.71 ± 0.05**
DUSP6	**0.62 ± 0.06**	**0.61 ± 0.07**	**0.63 ± 0.09**	**0.72 ± 0.06**	**0.72 ± 0.09**	**0.73 ± 0.07**

Data represent mean fold changes ± SEM for all participants together (*n* = 24) or for separate groups (*n* = 11, controls; *n* = 13, dysglycemics); *P*‐values were calculated by paired Student's *t*‐test, *P* < 0.05 versus pre‐exercise (bold). Baseline pre‐exercise values are presented in Table 1. MKPs, MAPK phosphatases; DUSP, dual specificity phosphatase.

**Figure 3:**
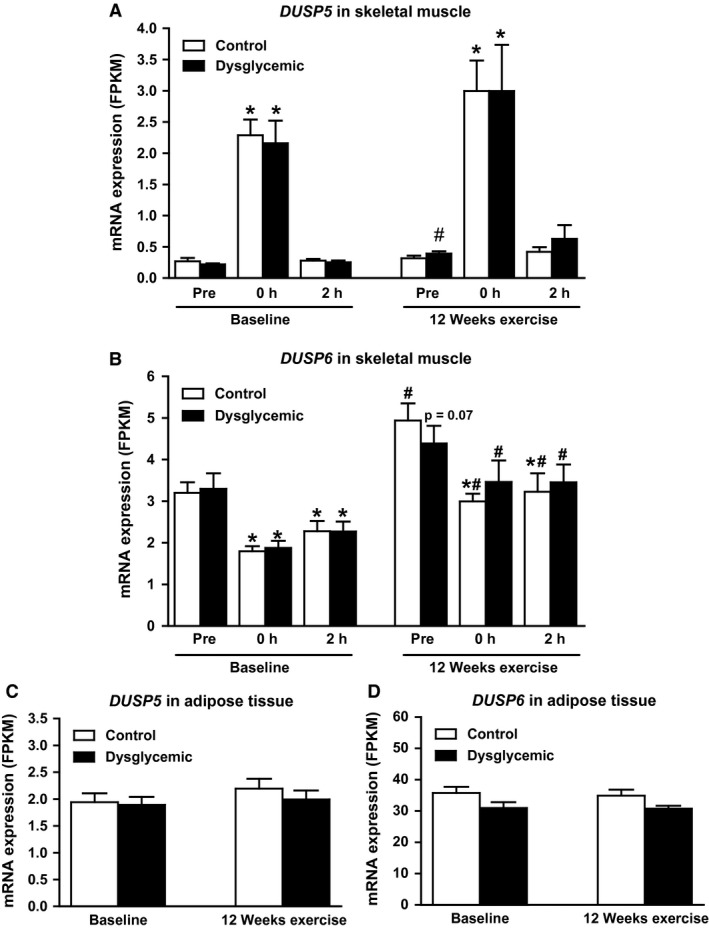
*DUSP5* and *DUSP6* expression in skeletal muscle and adipose tissue before and after long‐term exercise. Control (*n* = 13) and dysglycemic (*n* = 11) men performed an acute bout of exercise (45 min, 70% *V*O_2_max) at baseline and after 12 weeks of exercise. Skeletal muscle biopsies (A and B) were taken before (pre), immediately after (0 h), and 2 h after the acute bout. Adipose tissue biopsies (C and D) were taken 30–60 min after exercise. mRNA expression of *DUSP5* (A and C) and *DUSP6* (B and D) was assessed by RNA‐seq. Data represent mean FPKM ± SEM;* P*‐values were calculated by paired Student's *t*‐test. *P <* *0.05 versus pre, ^#^ *P *<* *0.05 versus corresponding value at baseline.

*DUSP5* and *DUSP6* displayed the most striking pattern of alteration by acute exercise in skeletal muscle, whereas no change was observed in adipose tissue (Fig. 3). At baseline, *DUSP5* mRNA expression increased 9.1‐fold just after exercise and returned to the pre‐exercise level within 2 h. *DUSP6* expression was oppositely regulated and decreased by 43% immediately after exercise, and the mRNA expression was still 30% reduced compared to the pre‐exercise expression after 2 h recovery. We observed no difference in pre‐exercise mRNA expression and regulation of *DUSP5* and *DUSP6* by acute exercise between control and dysglycemic men at baseline (Table 1; Fig. 3A and B). After 12 weeks of training, pre‐exercise *DUSP5* mRNA expression was significantly increased 1.8‐fold in the dysglycemic group (Fig. 3A; Table 3), whereas the pre‐exercise mRNA expression of *DUSP6* was significantly increased 1.5‐fold in the control group (Fig. 3B; Table 3). Acute response to exercise remained unchanged in the dysglycemic group following 12 weeks of exercise intervention. Moreover, there were no significant changes in the expression of MKPs in adipose tissue after 12 weeks of exercise (Table 3).

**Table 3:** Regulation mRNA expression of MKPs in skeletal muscle and adipose tissue in relation to long‐term exercise

MKP	Skeletal muscle			Adipose tissue		
	All	Control	Dysglycemic	All	Control	Dysglycemic
DUSP5	**1.76 ± 0.24**	1.67 ± 0.41	**1.88 ± 0.20**	1.16 ± 0.08	1.19 ± 0.11	1.12 ± 0.13
DUSP4	1.62 ± 0.30	2.01 ± 0.48	1.15 ± 0.27	1.23 ± 0.15	1.14 ± 0.17	1.34 ± 0.25
DUSP6	**1.58 ± 0.14**	**1.68 ± 0.20**	1.46 ± 0.22	1.01 ± 0.04	0.99 ± 0.05	1.03 ± 0.07
DUSP10	**1.37 ± 0.10**	**1.50 ± 0.12**	1.21 ± 0.17	1.14 ± 0.07	1.21 ± 0.11	1.06 ± 0.09
DUSP2	1.17 ± 0.12	1.12 ± 0.17	1.23 ± 0.18	1.21 ± 0.19	1.15 ± 0.26	1.27 ± 0.28
DUSP1	1.11 ± 0.18	1.26 ± 0.31	0.93 ± 0.12	1.18 ± 0.10	1.08 ± 0.13	1.31 ± 0.16
DUSP8	1.09 ± 0.08	1.12 ± 0.15	1.05 ± 0.06	1.18 ± 0.09	1.25 ± 0.14	1.09 ± 0.09
DUSP16	1.05 ± 0.04	**1.11 ± 0.04**	0.99 ± 0.08	1.08 ± 0.04	1.07 ± 0.06	1.09 ± 0.06
DUSP7	0.98 ± 0.05	0.96 ± 0.09	1.02 ± 0.06	0.97 ± 0.04	0.94 ± 0.05	0.99 ± 0.06

Data represent mean fold changes ± SEM for all participants together (*n* = 24) or for separate groups (*n* = 11, controls; *n* = 13, dysglycemic subjects); *P*‐values were calculated by paired Student's *t*‐test, *P* < 0.05 versus baseline (bold). MKPs, MAPK phosphatases; DUSP, dual specificity phosphatase.

### Expression of *DUSP5* and *DUSP6* in primary skeletal muscle cells

**Figure 4:**
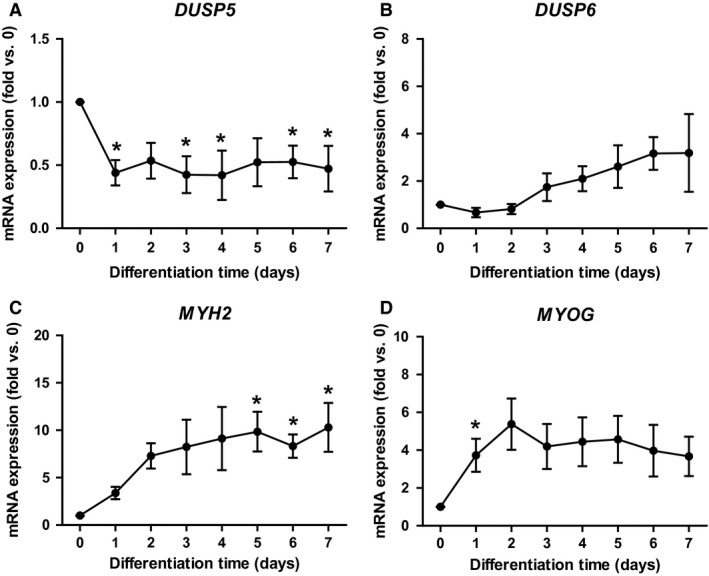
*DUSP5* and *DUSP6* mRNA expression in primary cultures of human myotubes. Primary human myoblasts from four donors were seeded, and differentiation was initiated at 80% confluency. RNA was harvested before start of the differentiation and at indicated time points. mRNA expression of *DUSP5* (A), *DUSP6* (B), and differentiation markers *MYH2* (C) and *MYOG* (D) were monitored by qPCR and normalized to *RPLP0* expression. Data represent means ± SEM;* P*‐values were calculated by one‐way ANOVA. *P <* *0.05 versus day 0 of differentiation. DUSP, dual specificity phosphatase; qPCR, quantitative real‐time PCR; RPLP0, ribosomal protein P0.

Primary human skeletal muscle cells were used as an in vitro model, and the mRNA expression of *DUSP5* and *DUSP6* was monitored during myogenic differentiation (Fig. 4). *DUSP5* mRNA expression decreased on the first day of differentiation, which probably is related to the change of growth medium containing 10% FBS and several growth factors to differentiation medium containing 2% horse serum with no growth factors. During the following 6 days, no change in *DUSP5* mRNA expression was observed. *DUSP6* mRNA expression increased ~3.5‐fold during differentiation. The proper differentiation of myotubes was monitored by assessing mRNA expression of the myogenic markers *MYH2* encoding MHCIIa, which was increased 10‐fold (Fig. 4C), and *MYOG*, which was increased fourfold (Fig. 4D).

### mRNA expression of *DUSP5* and *DUSP6* was regulated by Dex

**Figure 5:**
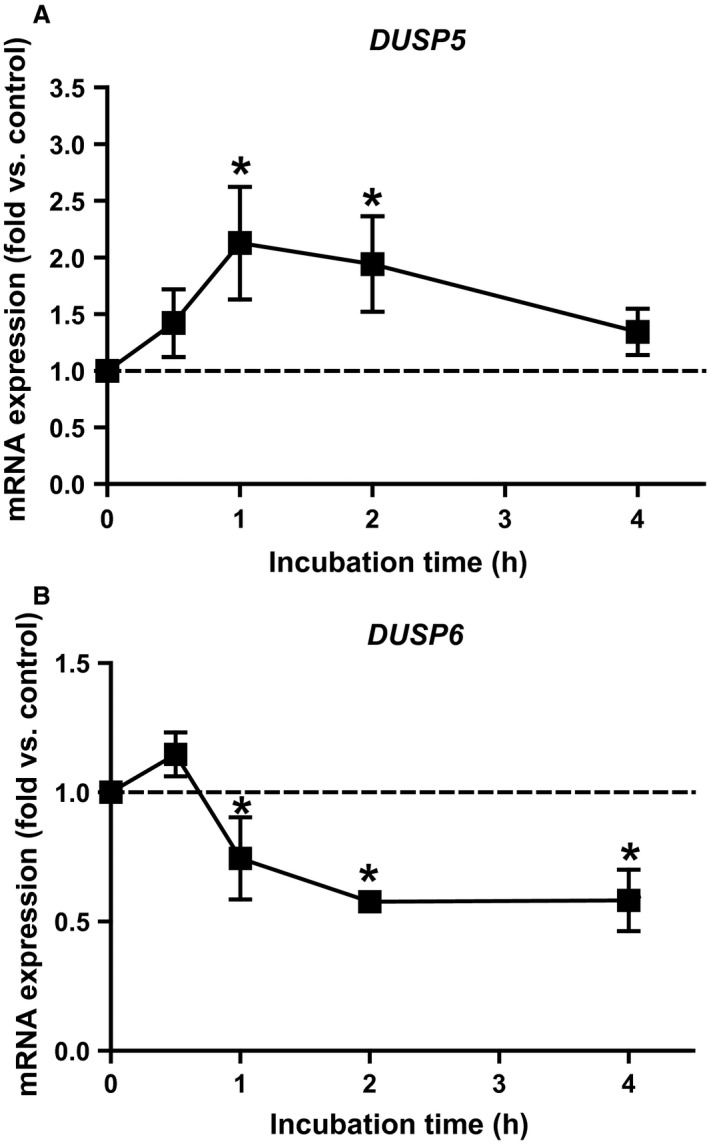
Effect of Dex on *DUSP5* and *DUSP6* mRNA expression. At day 6 of differentiation, primary human myotubes were incubated with 0.5 *μ*mol/L Dex, and expression of *DUSP5* (A) and *DUSP6* (B) was monitored by qPCR. Data represent means ± SEM;* P*‐values were calculated by one‐way ANOVA. *P <* *0.05 versus 0 h, *n* ≥ 3. Dex, dexamethasone; DUSP, dual specificity phosphatase; qPCR, quantitative real‐time PCR.

Plasma cortisol levels increase during acute exercise (Duclos et al. 2003) as also observed in our study (Norheim et al. 2014). To investigate a potential link between changes in plasma cortisol concentration and the regulation of *DUSP5* and *DUSP6* mRNA expression during acute exercise, we incubated primary human myotubes with 0.5 *μ*mol/L Dex for 15–240 min. *DUSP5* mRNA expression increased 2.1‐fold after 1 h and returned to basal level after 4 h (Fig. 5A). *DUSP6* mRNA expression was reduced by 25% after 1 h and by 40% after 2–4 h Dex exposure (Fig. 5B). However, there was no correlation between the change in plasma cortisol during the acute exercise bout and the change of expression of either MKP (data not shown).

### Induction of *DUSP5* mRNA expression occurs via ERK1/2

**Figure 6:**
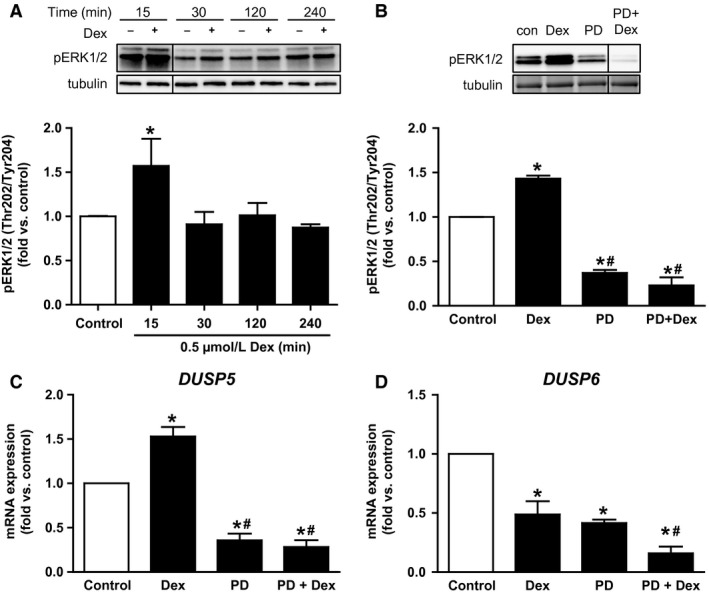
Dex induced ERK1/2 phosphorylation, which was required for mRNA expression of *DUSP5* and *DUSP6*. (A) Primary human myotubes were incubated with 0.5 *μ*mol/L Dex for 15–240 min and phosphorylation of ERK1/2 was monitored. (B) Cells were incubated with 10 *μ*mol/L PD for 1 h prior to incubation with Dex (15 min), and phosphorylation of ERK1/2 was monitored. (C) *DUSP5* and *DUSP6* (D) mRNA expression were monitored by qPCR after exposure to PD (1 h), Dex (1 h) and a combination of both, respectively. Data represent means ± SEM;* P*‐values were calculated by one‐way ANOVA. *P <* *0.05 versus control, ^#^ *P *<* *0.05 versus Dex, *n* ≥ 3. Dex, dexamethasone; ERK, extracellular signal‐regulated kinase; DUSP, dual specificity phosphatase; PD, MEK inhibitor PD98059; qPCR, quantitative real‐time PCR.

Myotubes were incubated 15–240 min with Dex to assess the effect on ERK1/2 phosphorylation. As shown in Figure 6A, phosphorylation of ERK1/2 was increased after 15 min of Dex incubation (1.6‐fold, *P* = 0.01), and returned to basal level within 30 min. The MEK inhibitor PD98059 (PD) reduced the basal phosphorylation of ERK1/2 by 63%. Preincubation of myotubes with PD for 1 h followed by 15 min Dex exposure completely abolished phosphorylation of ERK1/2 (Fig. 6B), whereas total protein level of ERK1/2 was unaffected (data not shown).

Monitoring the effect of PD on mRNA expression of both MKPs in myotubes revealed that inhibiting ERK1/2 activity caused a reduction of *DUSP5* mRNA expression by 65% (Fig. 6C), which was not restored by 1 h administration of Dex in addition to PD. Moreover, 1 h incubation of myotubes with PD reduced the mRNA expression of *DUSP6* by 60% to a similar extent as Dex (Fig. 6D). Combining PD and Dex further reduced the mRNA expression of *DUSP6* to 16% of control cells.

## Discussion

### Regulation of *DUSP5* and *DUSP6* mRNA expression

We observed that after acute endurance exercise *DUSP5* was the most enhanced, whereas *DUSP6* was the most downregulated MKP among *DUSPs* expressed in human skeletal muscle (Table 2). Moreover, short‐term incubation of myotubes with Dex could mimic this pattern in vitro (Fig. 5). We further demonstrated that induction of *DUSP5* depended on ERK1/2 signaling.

Using microglia, Ham et al. (2015) showed that incubation with LPS induced *DUSP5* and repressed *DUSP6* mRNA expression, which is comparable to our results. Other scientists reported on induction of *DUSP5* mRNA expression in different mammalian cells by serum, heat shock, and vascular endothelial growth factor (Ishibashi et al. 1994; Kwak and Dixon 1995; Bellou et al. 2009; Kucharska et al. 2009). For *DUSP6*, fibroblast growth factor (Li et al. 2007; Ekerot et al. 2008), platelet‐derived growth factor (Jurek et al. 2009), nerve growth factor (Camps et al. 1998), and hypoxia (Bermudez et al. 2011) have been reported to induce its expression. However, we are the first to show that Dex induces *DUSP5* while decreasing *DUSP6* mRNA expression in primary human myotubes. Although we observed a significant increase in plasma cortisol after exercise as expected (Norheim et al. 2014), this increase did not correlate with the changes observed in the mRNA expression of *DUSP5* and *DUSP6* in skeletal muscle biopsies. An explanation might relate to differences in the dynamics of cortisol action in vivo and in vitro, differences between different glucocorticoids, or unknown factors. Our result on *DUSP6* repression is in contrast to a study in rat hepatoma cells where Dex incubation enhanced *DUSP6* mRNA expression after 6 h (Xu et al. 2005). The discrepancy might be explained by the use of different cell types (liver vs. skeletal muscle), and differences between cancer cell lines and primary cells. The effect of glucocorticoids on MKPs is not well studied except for DUSP1, which is induced by Dex in different cell types (Kassel et al. 2001; Lasa et al. 2002; Clark et al. 2008; Shah et al. 2014).

Induction of *DUSP5* mRNA expression in skeletal muscle cells occurred via activation of ERK1/2 as shown by the use of PD. This finding is supported by studies in other cell types like NIH 3T3, HeLa, and COS‐1 cells (Mandl et al. 2005; Kucharska et al. 2009). Kucharska et al. reported that a serum‐induced increase of *DUSP5* mRNA expression was prevented by the MEK inhibitor U0126. They also showed that signaling pathways involving JNK, p38 MAPK, or PI3K were not involved in the induction of *DUSP5* by serum (Kucharska et al. 2009). ERK1/2 phosphorylates mitogen‐ and stress‐activated kinases 1 and 2 as well as p90 ribosomal S6 kinase. These kinases may act on different transcription factors including Elk‐1. A recent study by Buffet et al. (2015) revealed that Elk‐1 is involved in the transcriptional control of *DUSP5*, providing a link between ERK1/2 activation, induction of *DUSP5*, and consequently an inhibition of ERK1/2 activity.

Interestingly, we observed that incubating myotubes with PD reduced *DUSP6* mRNA expression along with decreasing basal ERK1/2 phosphorylation. This observation has been reported also in other studies (Bermudez et al. 2011; Ham et al. 2015) and may suggest that a certain ERK1/2 activity is required to maintain the basal level of DUSP6. Moreover, we found that combining PD and Dex further reduced the mRNA expression of *DUSP6* compared to both PD and Dex alone. Hence, we suggest that the effect of Dex on *DUSP6* mRNA expression may be independent of its effect on ERK1/2 activity.

### Potential interaction between ERK1/2 activity and DUSP5/DUSP6 during exercise

**Figure 7:**
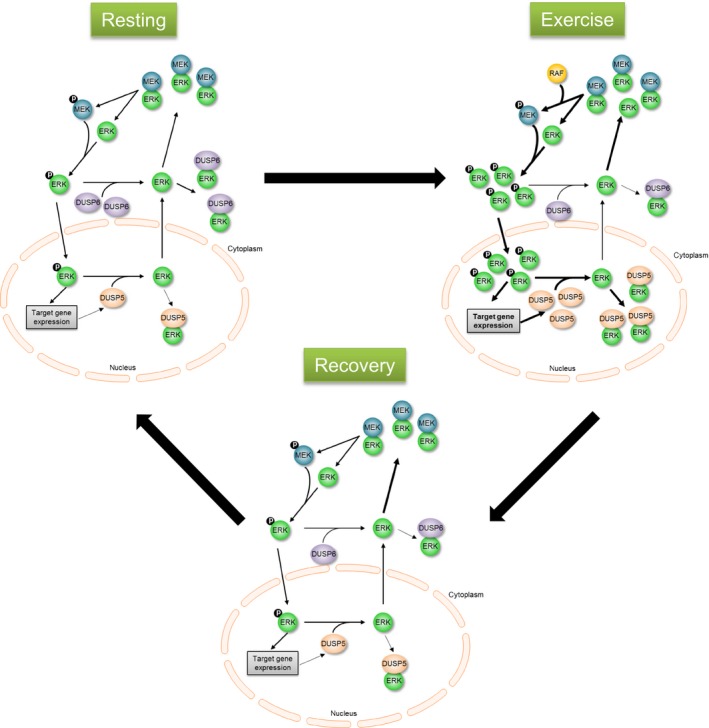
Hypothetical interplay between ERK1/2 signaling, DUSP5, and DUSP6 in skeletal muscle before and after exercise. Before exercise, basal phosphorylation of MEK and ERK1/2 is low. Inactive ERK1/2 is bound to inactive MEK and DUSP6 in cytoplasm (resting). DUSP6 is expressed at higher levels than DUSP5. During exercise MEK is activated by RAF leading to increased phosphorylation of ERK1/2, which is translocated into the nucleus and increases ERK1/2 target gene expression including DUSP5 (exercise). Enhanced DUSP5 increases dephosphorylation and trapping of ERK1/2 in the nucleus, and less ERK1/2 is recycled to cytoplasm. A higher proportion of cytoplasmic ERK1/2 is available for phosphorylation by MEK due to reduced level of DUSP6 (exercise). During recovery, MEK and ERK1/2 activities are reduced to basal level, normalizing the DUSP5 level. ERK1/2 is translocated back to the cytoplasm and most of it is bound to MEK, whereas DUSP6 level is still low (recovery). ERK, extracellular signal‐regulated kinase; DUSP, dual specificity phosphatase; MEK, MAPK kinase.

Acute endurance exercise stimulates MAPK pathways in skeletal muscle including ERK1/2 and its upstream regulators Raf‐1 and MEK (Aronson et al. 1997; Widegren et al. 2001). Widegren et al. (1998) observed maximum activity of ERK1/2 after 30 min of exercise. After 60 min of exercise, phosphorylation of ERK1/2 was slightly reduced, and after cessation of exercise, ERK1/2 activity was restored to pre‐exercise level within 60 min. Increasing ERK1/2 activity promotes enhanced *DUSP5* mRNA expression (Fig. 7). DUSP5 dephosphorylates and sequesters inactive ERK in the nucleus, which might explain the slightly reduced ERK1/2 phosphorylation observed after 60 min of exercise. The exercise‐induced activation of the ERK1/2 pathway stops when exercise is terminated, whereas *DUSP5* is still highly expressed. In our study, we found ninefold higher *DUSP5* mRNA expression just after exercise compared to pre‐exercise expression. This suggests that restoration of pre‐exercise level of ERK1/2 phosphorylation within short recovery time may involve DUSP5 activity. Unfortunately, no sample material from our study was left to study phosphorylation of ERK1/2 and protein level of DUSP5 and DUSP6 after acute exercise. Hence, we do not know whether the regulation of *DUSP5* and *DUSP6* mRNA expression by acute exercise is followed by corresponding alterations of the protein level. However, others reported that changes in mRNA level of *DUSP5* and *DUSP6* resulted in corresponding alterations of protein level (Nunes‐Xavier et al. 2010; Ham et al. 2015). After 2 h recovery, *DUSP5* mRNA expression was restored to its pre‐exercise level because ERK1/2 activity was also on its pre‐exercise level (Fig. 7). Thus, DUSP5 may play an important role in controlling nuclear ERK1/2 signaling in a stimulus‐ and time‐dependent manner.

Although acute exercise induced *DUSP5* mRNA expression, we observed that mRNA expression of *DUSP6* was reduced immediately after and 2 h after exercise. This difference in kinetics compared to *DUSP5* was also observed in vitro. As mentioned earlier, DUSP6 dephosphorylates active ERK1/2 and binds to inactive ERK1/2 in the cytoplasm thereby competing with MEK, the upstream kinase of ERK1/2. Hence, a sustained reduction of DUSP6 may increase the relative amount of ERK1/2 available for phosphorylation by MEK, which may promote ERK1/2 activation in the cytoplasm and may help to restore the basal ERK1/2 activity.

### Physiological impact of DUSP5 and DUSP6

The physiological role of DUSP5 in skeletal muscle in the context of exercise is largely unexplored. We hypothesize that its role in the tight regulation of ERK1/2 signaling might be related to the regulation of fatty acid metabolism. This assumption is based on studies showing that inhibition of ERK1/2 prevented contraction‐induced translocation of fatty acid translocase (FAT/CD36) to the plasma membrane (Turcotte et al. 2005; Raney and Turcotte 2007). Thus, uptake of fatty acids during contraction was not increased when ERK1/2 activity was blocked. Moreover, contraction‐induced activation of ERK1/2 increased fatty acid oxidation, which was reduced by inhibition of ERK1/2 (Raney and Turcotte 2007). FAT/CD36 is also present in mitochondrial membranes (Campbell et al. 2004; Bezaire et al. 2006), and the mitochondrial FAT/CD36 protein content was increased by muscle contraction in parallel with enhanced fatty acid oxidation in wild‐type but not FAT/CD36 knockout mice (Holloway et al. 2009). These and other studies suggest a key role for FAT/CD36 in the exercise‐induced increase of fatty acid uptake and oxidation (Yoshida et al. 2013), which is regulated by ERK1/2 signaling. Hence, DUSP5‐mediated deactivation of ERK1/2 signaling after exercise might be important to prevent excessive uptake of fatty acids by limiting high ERK1/2 activity to periods of muscle activity.

Bonen et al. observed a permanent relocation of FAT/CD36 to the sarcolemma in skeletal muscle of type 2 diabetic patients leading to a marked increase in skeletal muscle fatty acid transport (Bonen et al. 2004; Aguer et al. 2010). Lipid oversupply may lead to the accumulation of lipids interfering with insulin signaling as observed in type 2 diabetic and obese individuals (Holloway et al. 2010). Thus, the higher pre‐exercise *DUSP5* mRNA expression after 12 weeks exercise in dysglycemic subjects may reflect an adaptation to exercise, which is required to further impede FAT/CD36 translocation to sarcolemma by deactivating ERK1/2. This may reduce lipid supply to skeletal muscle and may improve insulin sensitivity in the dysglycemic group, which is a well‐known effect of exercise.

Also for DUSP6, the current knowledge on its impact on skeletal muscle is rather limited. One study investigating skeletal muscle regeneration identified DUSP6 as target of the homeo domain transcription factor Six1, which is expressed in satellite cells (Le Grand et al. 2012). These stem cells mediate skeletal muscle growth and regeneration, for example, after exercise and injury. Six1 is important to ensure replenishment of the stem cell pool. By controlling *DUSP6* mRNA expression, Six1 may control the duration of ERK1/2 signaling during muscle regeneration. DUSP6 has been studied more extensively in relation to metabolism. Genetic or diet‐induced obese mice display elevated hepatic DUSP6 expression (Xu et al. 2005; Wu et al. 2010). DUSP6 promotes gluconeogenic gene transcription and is involved in the regulation of hepatic gluconeogenesis (Xu et al. 2005; Wu et al. 2010). Moreover, long‐term exercise in mice reduced hepatic DUSP6 expression and suppressed gluconeogenesis (Souza Pauli et al. 2014). However, other functions of DUSP6 in skeletal muscle, in particular during exercise, remain elusive, and further studies are needed to clarify the impact of our observations.

## Conclusion

Our study shows that acute exercise affects mRNA expression of the two ERK1/2‐specific MKPs *DUSP5* and *DUSP6* in human skeletal muscle, but not in adipose tissue. This may suggest a potential role for DUSP5 and DUSP6 in controlling exercise‐activated ERK1/2 signaling in human skeletal muscle.

## Ethics approval and consent to participate

Our study adhered to the Declaration of Helsinki and was approved by the National Regional Committee for Medical and Health Research Ethics North, Tromsø, Oslo, Norway. Written informed consent was obtained from all participants prior to any study‐related procedure.

## Conflict of Interest

None declared.

## References

[phy213459-bib-0001] Aguer, C. , J. Mercier , C. Y. Man , L. Metz , S. Bordenave , K. Lambert , et al. 2010 Intramyocellular lipid accumulation is associated with permanent relocation ex vivo and in vitro of fatty acid translocase (FAT)/CD36 in obese patients. Diabetologia 53:1151–1163. 20333349 10.1007/s00125-010-1708-x

[phy213459-bib-0002] Arkell, R. S. , R. J. Dickinson , M. Squires , S. Hayat , S. M. Keyse , and S. J. Cook . 2008 DUSP6/MKP‐3 inactivates ERK1/2 but fails to bind and inactivate ERK5. Cell. Signal. 20:836–843. 18280112 10.1016/j.cellsig.2007.12.014

[phy213459-bib-0003] Aronson, D. , M. A. Violan , S. D. Dufresne , D. Zangen , R. A. Fielding , and L. J. Goodyear . 1997 Exercise stimulates the mitogen‐activated protein kinase pathway in human skeletal muscle. J. Clin. Invest. 99:1251–1257. 9077533 10.1172/JCI119282 PMC507939

[phy213459-bib-0004] Bellou, S. , M. A. Hink , E. Bagli , E. Panopoulou , P. I. Bastiaens , C. Murphy , et al. 2009 VEGF autoregulates its proliferative and migratory ERK1/2 and p38 cascades by enhancing the expression of DUSP1 and DUSP5 phosphatases in endothelial cells. Am. J. Physiol. Cell Physiol. 297:C1477–C1489. 19741200 10.1152/ajpcell.00058.2009

[phy213459-bib-0005] Bermudez, O. , P. Jouandin , J. Rottier , C. Bourcier , G. Pages , and C. Gimond . 2011 Post‐transcriptional regulation of the DUSP6/MKP‐3 phosphatase by MEK/ERK signaling and hypoxia. J. Cell. Physiol. 226:276–284. 20665674 10.1002/jcp.22339

[phy213459-bib-0006] Bezaire, V. , C. R. Bruce , G. J. Heigenhauser , N. N. Tandon , J. F. Glatz , J. J. Luiken , et al. 2006 Identification of fatty acid translocase on human skeletal muscle mitochondrial membranes: essential role in fatty acid oxidation. Am. J. Physiol. Endocrinol. Metab. 290:E509–E515. 16219667 10.1152/ajpendo.00312.2005

[phy213459-bib-0007] Bonen, A. , M. L. Parolin , G. R. Steinberg , J. Calles‐Escandon , N. N. Tandon , J. F. Glatz , et al. 2004 Triacylglycerol accumulation in human obesity and type 2 diabetes is associated with increased rates of skeletal muscle fatty acid transport and increased sarcolemmal FAT/CD36. FASEB J. 18:1144–1146. 15132977 10.1096/fj.03-1065fje

[phy213459-bib-0008] Buffet, C. , M. G. Catelli , K. Hecale‐Perlemoine , L. Bricaire , C. Garcia , A. Gallet‐Dierick , et al. 2015 Dual specificity phosphatase 5, a specific negative regulator of ERK signaling, is induced by serum response factor and Elk‐1 transcription factor. PLoS One 10:e0145484. 26691724 10.1371/journal.pone.0145484 PMC4687125

[phy213459-bib-0009] Campbell, S. E. , N. N. Tandon , G. Woldegiorgis , J. J. Luiken , J. F. Glatz , and A. Bonen . 2004 A novel function for fatty acid translocase (FAT)/CD36: involvement in long chain fatty acid transfer into the mitochondria. J. Biol. Chem. 279:36235–36241. 15161924 10.1074/jbc.M400566200

[phy213459-bib-0010] Camps, M. , C. Chabert , M. Muda , U. Boschert , C. Gillieron , and S. Arkinstall . 1998 Induction of the mitogen‐activated protein kinase phosphatase MKP3 by nerve growth factor in differentiating PC12. FEBS Lett. 425:271–276. 9559664 10.1016/s0014-5793(98)00250-6

[phy213459-bib-0011] Camps, M. , A. Nichols , and S. Arkinstall . 2000 Dual specificity phosphatases: a gene family for control of MAP kinase function. FASEB J. 14:6–16. 10627275

[phy213459-bib-0012] Catenacci, V. A. , and H. R. Wyatt . 2007 The role of physical activity in producing and maintaining weight loss. Nat. Clin. Pract. Endocrinol. Metab. 3:518–529. 17581621 10.1038/ncpendmet0554 PMC4578965

[phy213459-bib-0013] Clark, A. R. , J. R. Martins , and C. R. Tchen . 2008 Role of dual specificity phosphatases in biological responses to glucocorticoids. J. Biol. Chem. 283:25765–25769. 18541529 10.1074/jbc.R700053200 PMC3258850

[phy213459-bib-0014] Duclos, M. , C. Gouarne , and D. Bonnemaison . 2003 Acute and chronic effects of exercise on tissue sensitivity to glucocorticoids. J. Appl. Physiol. (1985) 94:869–875. 12433870 10.1152/japplphysiol.00108.2002

[phy213459-bib-0015] Egan, B. , and J. R. Zierath . 2013 Exercise metabolism and the molecular regulation of skeletal muscle adaptation. Cell Metab. 17:162–184. 23395166 10.1016/j.cmet.2012.12.012

[phy213459-bib-0016] Ekerot, M. , M. P. Stavridis , L. Delavaine , M. P. Mitchell , C. Staples , D. M. Owens , et al. 2008 Negative‐feedback regulation of FGF signalling by DUSP6/MKP‐3 is driven by ERK1/2 and mediated by Ets factor binding to a conserved site within the DUSP6/MKP‐3 gene promoter. Biochem. J. 412:287–298. 18321244 10.1042/BJ20071512 PMC2474557

[phy213459-bib-0017] Groom, L. A. , A. A. Sneddon , D. R. Alessi , S. Dowd , and S. M. Keyse . 1996 Differential regulation of the MAP, SAP and RK/p38 kinases by Pyst1, a novel cytosolic dual‐specificity phosphatase. EMBO J. 15:3621–3632. 8670865 PMC451978

[phy213459-bib-0018] Ham, J. E. , E. K. Oh , D. H. Kim , and S. H. Choi . 2015 Differential expression profiles and roles of inducible DUSPs and ERK1/2‐specific constitutive DUSP6 and DUSP7 in microglia. Biochem. Biophys. Res. Commun. 467:254–260. 26435497 10.1016/j.bbrc.2015.09.180

[phy213459-bib-0019] Haugen, F. , F. Norheim , H. Lian , A. J. Wensaas , S. Dueland , O. Berg , et al. 2010 IL‐7 is expressed and secreted by human skeletal muscle cells. Am. J. Physiol. Cell Physiol. 298:C807–C816. 20089933 10.1152/ajpcell.00094.2009

[phy213459-bib-0020] Hawley, J. A. , M. Hargreaves , M. J. Joyner , and J. R. Zierath . 2014 Integrative biology of exercise. Cell 159:738–749. 25417152 10.1016/j.cell.2014.10.029

[phy213459-bib-0022] Holloway, G. P. , S. S. Jain , V. Bezaire , X. X. Han , J. F. Glatz , J. J. Luiken , et al. 2009 FAT/CD36‐null mice reveal that mitochondrial FAT/CD36 is required to upregulate mitochondrial fatty acid oxidation in contracting muscle. Am. J. Physiol. Regul. Integr. Comp. Physiol. 297:R960–R967. 19625692 10.1152/ajpregu.91021.2008

[phy213459-bib-0023] Holloway, G. P. , R. W. Schwenk , J. J. F. P. Luiken , J. F. C. Glatz , and A. Bonen . 2010 Fatty acid transport in skeletal muscle: role in energy provision and insulin resistance. Clin. Lipidol. 5:731–745.

[phy213459-bib-0024] Ishibashi, T. , D. P. Bottaro , P. Michieli , C. A. Kelley , and S. A. Aaronson . 1994 A novel dual specificity phosphatase induced by serum stimulation and heat shock. J. Biol. Chem. 269:29897–29902. 7961985

[phy213459-bib-0025] Jurek, A. , K. Amagasaki , A. Gembarska , C. H. Heldin , and J. Lennartsson . 2009 Negative and positive regulation of MAPK phosphatase 3 controls platelet‐derived growth factor‐induced Erk activation. J. Biol. Chem. 284:4626–4634. 19106095 10.1074/jbc.M808490200

[phy213459-bib-0026] Karlsson, M. , J. Mathers , R. J. Dickinson , M. Mandl , and S. M. Keyse . 2004 Both nuclear‐cytoplasmic shuttling of the dual specificity phosphatase MKP‐3 and its ability to anchor MAP kinase in the cytoplasm are mediated by a conserved nuclear export signal. J. Biol. Chem. 279:41882–41891. 15269220 10.1074/jbc.M406720200

[phy213459-bib-0027] Kassel, O. , A. Sancono , J. Kratzschmar , B. Kreft , M. Stassen , and A. C. Cato . 2001 Glucocorticoids inhibit MAP kinase via increased expression and decreased degradation of MKP‐1. EMBO J. 20:7108–7116. 11742987 10.1093/emboj/20.24.7108 PMC125780

[phy213459-bib-0028] Kim, M. , K. H. Lee , S. W. Yoon , B. S. Kim , J. Chun , and H. Yi . 2013 Analytical tools and databases for metagenomics in the next‐generation sequencing era. Genomics Inform. 11:102–113. 24124405 10.5808/GI.2013.11.3.102 PMC3794082

[phy213459-bib-0029] Knowler, W. C. , E. Barrett‐Connor , S. E. Fowler , R. F. Hamman , J. M. Lachin , E. A. Walker , et al. 2002 Reduction in the incidence of type 2 diabetes with lifestyle intervention or metformin. N. Engl. J. Med. 346:393–403. 11832527 10.1056/NEJMoa012512 PMC1370926

[phy213459-bib-0030] Krook, A. , U. Widegren , X. J. Jiang , J. Henriksson , H. Wallberg‐Henriksson , D. Alessi , et al. 2000 Effects of exercise on mitogen‐ and stress‐activated kinase signal transduction in human skeletal muscle. Am. J. Physiol. Regul. Integr. Comp. Physiol. 279:R1716–R1721. 11049854 10.1152/ajpregu.2000.279.5.R1716

[phy213459-bib-0031] Kucharska, A. , L. K. Rushworth , C. Staples , N. A. Morrice , and S. M. Keyse . 2009 Regulation of the inducible nuclear dual‐specificity phosphatase DUSP5 by ERK MAPK. Cell. Signal. 21:1794–1805. 19666109 10.1016/j.cellsig.2009.07.015

[phy213459-bib-0032] Kwak, S. P. , and J. E. Dixon . 1995 Multiple dual specificity protein tyrosine phosphatases are expressed and regulated differentially in liver cell lines. J. Biol. Chem. 270:1156–1160. 7836374 10.1074/jbc.270.3.1156

[phy213459-bib-0800] Langleite, T. M. , J. Jensen , F. Norheim , H. L. Gulseth , D. S. Tangen , et al. 2016 Insulin sensitivity, body composition and adipose depots following 12 w combined endurance and strength training in dysglycemic and normoglycemic sedentary men. Arch. Physiol. Biochem. 122:167–179. 27477619 10.1080/13813455.2016.1202985

[phy213459-bib-0033] Langmead, B. , C. Trapnell , M. Pop , and S. L. Salzberg . 2009 Ultrafast and memory‐efficient alignment of short DNA sequences to the human genome. Genome Biol. 10:R25. 19261174 10.1186/gb-2009-10-3-r25 PMC2690996

[phy213459-bib-0034] Lasa, M. , S. M. Abraham , C. Boucheron , J. Saklatvala , and A. R. Clark . 2002 Dexamethasone causes sustained expression of mitogen‐activated protein kinase (MAPK) phosphatase 1 and phosphatase‐mediated inhibition of MAPK p38. Mol. Cell. Biol. 22:7802–7811. 12391149 10.1128/MCB.22.22.7802-7811.2002 PMC134716

[phy213459-bib-0035] Le Grand, F. , R. Grifone , P. Mourikis , C. Houbron , C. Gigaud , J. Pujol , et al. 2012 Six1 regulates stem cell repair potential and self‐renewal during skeletal muscle regeneration. J. Cell Biol. 198:815–832. 22945933 10.1083/jcb.201201050 PMC3432771

[phy213459-bib-0036] Li, C. , D. A. Scott , E. Hatch , X. Tian , and S. L. Mansour . 2007 Dusp6 (Mkp3) is a negative feedback regulator of FGF‐stimulated ERK signaling during mouse development. Development 134:167–176. 17164422 10.1242/dev.02701 PMC2424197

[phy213459-bib-0037] Li, Y. , S. Lee , T. Langleite , F. Norheim , S. Pourteymour , J. Jensen , et al. 2014 Subsarcolemmal lipid droplet responses to a combined endurance and strength exercise intervention. Physiol. Rep. 2:e12187. 25413318 10.14814/phy2.12187 PMC4255802

[phy213459-bib-0038] Mandl, M. , D. N. Slack , and S. M. Keyse . 2005 Specific inactivation and nuclear anchoring of extracellular signal‐regulated kinase 2 by the inducible dual‐specificity protein phosphatase DUSP5. Mol. Cell. Biol. 25:1830–1845. 15713638 10.1128/MCB.25.5.1830-1845.2005 PMC549372

[phy213459-bib-0039] Muda, M. , U. Boschert , R. Dickinson , J. C. Martinou , I. Martinou , M. Camps , et al. 1996 MKP‐3, a novel cytosolic protein‐tyrosine phosphatase that exemplifies a new class of mitogen‐activated protein kinase phosphatase. J. Biol. Chem. 271:4319–4326. 8626780 10.1074/jbc.271.8.4319

[phy213459-bib-0040] Norheim, F. , M. Hjorth , T. M. Langleite , S. Lee , T. Holen , C. Bindesboll , et al. 2014 Regulation of angiopoietin‐like protein 4 production during and after exercise. Physiol. Rep. 2:e12109. 25138789 10.14814/phy2.12109 PMC4246580

[phy213459-bib-0041] Nunes‐Xavier, C. E. , C. Tarrega , R. Cejudo‐Marin , J. Frijhoff , A. Sandin , A. Ostman , et al. 2010 Differential up‐regulation of MAP kinase phosphatases MKP3/DUSP6 and DUSP5 by Ets2 and c‐Jun converge in the control of the growth arrest versus proliferation response of MCF‐7 breast cancer cells to phorbol ester. J. Biol. Chem. 285:26417–26430. 20554528 10.1074/jbc.M110.121830 PMC2924073

[phy213459-bib-0042] Pedersen, B. K. , and B. Saltin . 2015 Exercise as medicine – evidence for prescribing exercise as therapy in 26 different chronic diseases. Scand. J. Med. Sci. Sports 25:1–72. 10.1111/sms.12581 26606383

[phy213459-bib-0043] Raney, M. A. , and L. P. Turcotte . 2007 Evidence for the regulation of contraction‐induced fatty acid oxidation via extracellular signal‐regulated kinase 1/2 activation independent of changes in fatty acid uptake. Metabolism 56:1192–1200. 17697861 10.1016/j.metabol.2007.04.014

[phy213459-bib-0044] Robinson, M. D. , D. J. McCarthy , and G. K. Smyth . 2010 edgeR: a Bioconductor package for differential expression analysis of digital gene expression data. Bioinformatics 26:139–140. 19910308 10.1093/bioinformatics/btp616 PMC2796818

[phy213459-bib-0045] Shah, S. , E. M. King , A. Chandrasekhar , and R. Newton . 2014 Roles for the mitogen‐activated protein kinase (MAPK) phosphatase, DUSP1, in feedback control of inflammatory gene expression and repression by dexamethasone. J. Biol. Chem. 289:13667–13679. 24692548 10.1074/jbc.M113.540799 PMC4036371

[phy213459-bib-0046] Souza Pauli, L. S. , E. C. Ropelle , C. T. de Souza , D. E. Cintra , A. S. da Silva , B. de Almeida Rodrigues , et al. 2014 Exercise training decreases mitogen‐activated protein kinase phosphatase‐3 expression and suppresses hepatic gluconeogenesis in obese mice. J. Physiol. 592:1325–1340. 24396063 10.1113/jphysiol.2013.264002 PMC3961090

[phy213459-bib-0047] Turcotte, L. P. , M. A. Raney , and M. K. Todd . 2005 ERK1/2 inhibition prevents contraction‐induced increase in plasma membrane FAT/CD36 content and FA uptake in rodent muscle. Acta Physiol. Scand. 184:131–139. 15916673 10.1111/j.1365-201X.2005.01445.x

[phy213459-bib-0048] Widegren, U. , X. J. Jiang , A. Krook , A. V. Chibalin , M. Bjornholm , M. Tally , et al. 1998 Divergent effects of exercise on metabolic and mitogenic signaling pathways in human skeletal muscle. FASEB J. 12:1379–1389. 9761781 10.1096/fasebj.12.13.1379

[phy213459-bib-0049] Widegren, U. , C. Wretman , A. Lionikas , G. Hedin , and J. Henriksson . 2000 Influence of exercise intensity on ERK/MAP kinase signalling in human skeletal muscle. Pflugers Arch. 441:317–322. 11211119 10.1007/s004240000417

[phy213459-bib-0050] Widegren, U. , J. W. Ryder , and J. R. Zierath . 2001 Mitogen‐activated protein kinase signal transduction in skeletal muscle: effects of exercise and muscle contraction. Acta Physiol. Scand. 172:227–238. 11472310 10.1046/j.1365-201x.2001.00855.x

[phy213459-bib-0051] Wu, Z. , P. Jiao , X. Huang , B. Feng , Y. Feng , S. Yang , et al. 2010 MAPK phosphatase‐3 promotes hepatic gluconeogenesis through dephosphorylation of forkhead box O1 in mice. J. Clin. Invest. 120:3901–3911. 20921625 10.1172/JCI43250 PMC2964981

[phy213459-bib-0052] Xu, H. , Q. Yang , M. Shen , X. Huang , M. Dembski , R. Gimeno , et al. 2005 Dual specificity MAPK phosphatase 3 activates PEPCK gene transcription and increases gluconeogenesis in rat hepatoma cells. J. Biol. Chem. 280:36013–36018. 16126724 10.1074/jbc.M508027200

[phy213459-bib-0053] Yoshida, Y. , S. S. Jain , J. T. McFarlan , L. A. Snook , A. Chabowski , and A. Bonen . 2013 Exercise‐ and training‐induced upregulation of skeletal muscle fatty acid oxidation are not solely dependent on mitochondrial machinery and biogenesis. J. Physiol. 591:4415–4426. 22890711 10.1113/jphysiol.2012.238451 PMC3784190

